# Comparative Genome Sequence Analysis Reveals the Extent of Diversity and Conservation for Glycan-Associated Proteins in *Burkholderia* spp.

**DOI:** 10.1155/2012/752867

**Published:** 2012-09-06

**Authors:** Hui San Ong, Rahmah Mohamed, Mohd Firdaus-Raih

**Affiliations:** School of Biosciences and Biotechnology, Faculty of Science and Technology, Universiti Kebangsaan Malaysia, 43600 Bangi, Malaysia

## Abstract

Members of the *Burkholderia* family occupy diverse ecological niches. In pathogenic family members, glycan-associated proteins are often linked to functions that include virulence, protein conformation maintenance, surface recognition, cell adhesion, and immune system evasion. Comparative analysis of available *Burkholderia* genomes has revealed a core set of 178 glycan-associated proteins shared by all *Burkholderia* of which 68 are homologous to known essential genes. The genome sequence comparisons revealed insights into species-specific gene acquisitions through gene transfers, identified an S-layer protein, and proposed that significantly reactive surface proteins are associated to sugar moieties as a potential means to circumvent host defense mechanisms. The comparative analysis using a curated database of search queries enabled us to gain insights into the extent of conservation and diversity, as well as the possible virulence-associated roles of glycan-associated proteins in members of the *Burkholderia* spp. The curated list of glycan-associated proteins used can also be directed to screen other genomes for glycan-associated homologs.

## 1. Introduction

Members of the genus *Burkholderia*, with over 30 known species, have a unique ability to occupy diverse ecological niches, ranging from soil to the human respiratory tract [1]. Several strains are known to enhance disease resistance in plants [2] and improve nitrogen fixation [3, 4]. *B. pseudomallei*, *B. mallei *and *B. cenocepacia *are known to be involved in lung infections and are well recognized as pathogens of humans and animals. *B. pseudomallei*, a soil dwelling member of the *Burkholderia *genus is the causative agent for melioidosis and is capable of existing as a latent infection for decades with the longest period reported being 62 years [5]. The current use of antimicrobial therapy to treat melioidosis patients often fails due to intrinsic resistance of these bacteria. Prior to the discovery of an RNA helicase inhibitor toxin [6], the pathogenicity and virulence factors associated to *B. pseudomallei* have been elusive and many reports were inconclusive. Despite this significant progress, much remains to be discovered regarding the virulence of *B. pseudomallei* and the *Burkholderia *pathogens in general, including potential roles played by glycan-associated proteins in pathogenesis. 

The interactions and association between proteins and carbohydrates play important roles in numerous cellular functions, including processes which are associated with the pathogenicity of many bacterial species. Proteins can interact with carbohydrate molecules covalently (glycoproteins) and non-covalently (protein-carbohydrate complexes). In bacteria, known roles of glycoproteins include recognition of immunodominant proteins during infection [7–9], animal/plant-microbe symbiosis [10], cell-cell interactions [11, 12] and evasion of the immune system [13]. Bacteria and viruses can efficiently adhere to the surface membranes of host cells through carbohydrate binding which then allows host invasion [14, 15]. This process is enhanced by the ability of carbohydrates to generate an array of structurally diverse moieties from a relatively small number of monosaccharide units [16]. Carbohydrates and carbohydrate derivatives are not only associated with the bacterial cellular architecture (e.g., capsule, lipopolysaccharide) but also function as information carriers [17], potential energy storage, and structural components. The diverse roles of carbohydrates result in the presence of carbohydrate molecules in various pathways responsible for systemic biological function of these bacteria.

In recent years, several genomes of *Burkholderia* species have been successfully sequenced. The completely sequenced *Burkholderia* genomes analyzed in this study are *B. pseudomallei *K96243 [18], *B. mallei *ATCC 23344 [19], *B. thailandensis *E264 [20], *B. xenovorans *LB400 [21], *B. phymatum *STM815 [22], *B. multivorans *ATCC 17616 [23], *B. ambifaria *MC40-6 [24], *B. vietnamiensis *G4 [25], and *B. cenocepacia *J2315 [26]. A number of experimental approaches have been carried out to identify glycan binding proteins present within a bacterial genome. However, the relatively smaller number of glycan-associated proteins identified through experimental methods barely reflects the actual amount of glycan-associated proteins present [27–29]. 

Here we report the inventorization and comparative analysis of glycan-associated proteins identified from the genomes of *Burkholderia *spp. This list comprises a wide array of biological functions ranging from involvement in cell envelope biogenesis, outer membrane pathway to the involvement in the transcription and translational machinery. In this work, we have compared and contrasted putative glycan-associated proteins by identifying both the orthologs as well as shared and unique glycan-associated proteins present in pathogenic and nonpathogenic *Burkholderia *spp. The selective pressure acting on glycan-associated surface proteins that showed reactivity during infection was also investigated.

## 2. Materials and Methods

### 2.1. Collection of Glycan-Associated Proteins in Prokaryotes

An initial target list of known prokaryotic glycan-associated proteins was compiled from the literature and keyword searches of the Uniprot Knowledgebase (Uniprot) [30] and Protein Data Bank (PDB) [31]. For this work, we define glycan-associated proteins as either glycoproteins or other proteins that are known to interact with glycans. The data collected from the literature reviews encompass the protein-carbohydrate complexes obtained through experimental techniques [27, 28] and proteins that have been reported to bind carbohydrates [32–34]. The searches in Uniprot and PDB were executed for the following keywords: glycoprotein, carbohydrate, sialic acid, glycan, bacteria glycoprotein, S-layer, polysaccharide, glucose, galactose, fructose, ribose, cellulose, heptose, trehalose, glucan, xylulose, rhamnose, capsule, mannose, maltose, and arabinose. The target list retrieved from the keyword searches was intensively curated and filtered manually for the proteins that have corresponding reports to be glycoproteins or associated to glycans either through the literature or as associated annotations in the databases searched such as a PDB protein structure with glycan bound.

### 2.2. Collection of Glycan-Associated Proteins in *Burkholderia* Species

This initial target list was used as queries for BLASTP [35] searches to extract *Burkholderia *spp. homologs. BLASTP searches were run using the default parameters set by the BLASTP program, with the exception of the *E*-value limited to 0.00001 [36]. The output of the BLASTP programs was divided into two categories: (i) sequences with significant alignment (*E*-value ≤ 0.00001) and (ii) sequences with *E*-values higher than 0.00001 (0.00001 < *E*-value ≤ 1) [37, 38]. The biological function of proteins that fall into the second category (0.00001 < *E*-value ≤ 1) was inspected using pathways presented in the Kyoto Encyclopaedia of Genes and Genomes (KEGG) database [39] and Pfam protein families database [40] searches to identify functional families and domains. If their biological properties were related to glycan, sugar, or carbohydrate binding, they were then regarded as glycan-associated proteins.

### 2.3. Identification of Orthologous Proteins in *Burkholderia* spp

The protein sequences for nine available *Burkholderia *genomes were downloaded from the National Center for Biotechnology Information (NCBI) website (http://ftp.ncbi.nih.gov/genomes/Bacteria/). Ortholog searches for these *Burkholderia* sequences were carried out using Inparanoid 4.1 package [41] with default parameters (overlap threshold = 0.5; confidence = 0.05; Matrix = BLOSUM45). Multiparanoid [42], an extension of the Inparanoid algorithms, was then used to cluster the orthologous groups from the nine *Burkholderia* species into a multispecies ortholog group. These outputs were then compiled into a dataset referred to as the *Burkholderia*_ortholog dataset. The BLASTP searches were carried out first followed by Inparanoid searches because not all complete genome sequence in prokaryote initial target list were available and the input for Inparanoid required the complete set of protein-encoding sequences.

### 2.4. Comparative Genome Analysis

Comparative analysis between *Burkholderia *spp. genomes was carried out using the Artemis Comparison Tool (ACT) [45] to detect conserved regions in different *Burkholderia* species. Comparison files between each *Burkholderia* species pairs were generated using BLASTN as an input for ACT.

### 2.5. Characterization of Glycan-Associated Proteins in *B. pseudomallei*

*B. pseudomallei *K96243 was chosen as the reference genome for further analysis due to its importance as a pathogen and the availability of the literature. Potential essential genes present in *B. pseudomallei *K96243 were identified via BLASTP searches [35], to *E*-value = 0.0001, other parameters at default settings, against the Database of Essential Genes [46]. Proteins with percentage identity above 40% [47, 48] were classified as potential essential genes for *B. pseudomallei*. The glycan-associated proteins identified were also subjected to PSORTb [49] to predict the subcellular location.

### 2.6. Sequence Analysis of Glycosylation Sites in Surface Proteins That Exhibit Significant Reactivity during Infection

Surface proteins identified by PSORTb [49] were subjected to glycosylation site prediction using EnsembleGly [50]. The physicochemical properties of these surface proteins were then subjected to a number of analyses, namely, (i) ProtParam [51] to calculate the protein isoelectric point; (ii) ProtScale (http://web.expasy.org/cgi-bin/protscale/protscale.pl) to generate hydrophobicity graph using Kyte and Doolittle hydropathic plot [52]; (iii) NetSurfP to calculate protein surface accessibility [53]; (iv) GOR IV [54] for the prediction of secondary structures; (v) SignalP version 3.0 [55] signal peptide prediction.

### 2.7. Measuring Selective Pressure on Surface Proteins

To investigate the selective pressure on these proteins, *K*_*a*_/*K*_*s*_ ratio (nonsynonymous substitution rate/synonymous substitution rate) between *B. pseudomallei* K96243 proteins with its orthologs was calculated using *K*_*a*_/*K*_*s*_ calculator v1.2 [56] by using the YN model [57]. A *K*_*a*_/*K*_*s*_ ratio significantly greater than one implies positive selection, less than one implies purifying selection, and a ratio of one indicates neutrality [56].

## 3. Results and Discussion

### 3.1. Compilation of Glycan-Associated Proteins in Prokaryotes

Keyword searches against the databases and the literature that were manually curated have successfully identified 1470 glycan-associated proteins across the kingdom Prokaryota. Of these 1470 glycan-associated proteins, 545 were previously reported in the literature while the remaining 925 glycan-associated proteins were identified through data mining of the Uniprot Knowledgebase [30] and the Protein Data Bank [31] as described in Section 2. The use of this curated collection of glycan-associated proteins to probe *Burkholderia* genomes has enabled the identification of conserved glycan-associated proteins as well as species-specific or unique protein sequences.

### 3.2. Compilation of Glycan-Associated Proteins Present in *Burkholderia *spp

**Figure 1:**
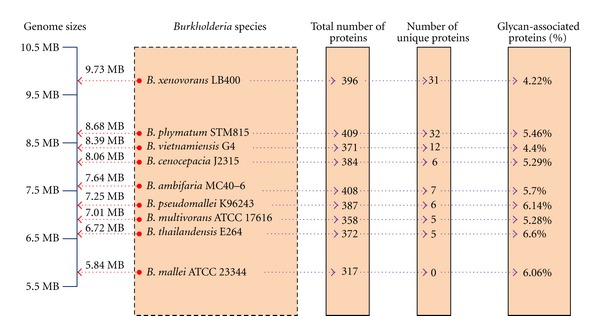
The total number of glycan-associated protein content found in nine *Burkholderia* species. The “total number of proteins” refers to total number of glycan-associated protein, and the “number of unique proteins” represent as the unique glycan-associated proteins that are found only in a specific species but does not have orthologs present in other *Burkholderia *spp. The percentages (%) of glycan-associated proteins were obtained by calculating the total number of glycan-associated proteins per the total number of protein coding genes.

The total number of glycan-associated protein content for each *Burkholderia* species analyzed is presented in Figure 1. From our analysis, the percentage of glycan-associated proteins measured against the number of protein-coding genes is not directly proportional to the total number of protein-coding genes or genome size. However, the reduced number of glycan-associated proteins and the absence of species unique glycan-associated proteins for the *B. mallei* ATCC 23344 proteome are perhaps a reflection of it having the smallest genome size (5.84 Mb) amongst the available genomes. Further investigation into the regions of the missing sequences relative to the *B. pseudomallei* glycan-associated gene clusters showed the presence of transposable genetic elements (Table 1). Transposable genetic elements are known to contribute to bacterial genome variability by causing transposition events [58]. This has formed the basis that *B. mallei *underwent high rates of gene deletion after divergence from a *B. pseudomallei *ancestor as part of the mechanism to remove transposase encoding genes thus resulting in the inadvertent deletion of the missing glycan-associated gene clusters.

### 3.3. Conserved Glycan-Associated Proteins in *Burkholderia* spp

The compilation of orthologs for glycan-associated proteins from the nine *Burkholderia* genomes, which was named the *Burkholderia*_ortholog dataset, yielded 178 glycan-associated proteins conserved across *Burkholderia* species, of which 68 are potential essential genes (Supplementary Table  1). The majority of these proteins have been annotated as proteins that are involved in the core regulation machinery such as translation and transcription (*bpss3220*, *bpsl3215)*, nucleotide excision repair (*bpss0058)*, proteolysis (*bpss1760*), and protein folding (*bpsl2827*).

Our analysis revealed no clear delineation of specific glycoproteins or glycan-associated proteins to only pathogenic members of *Burkholderia *spp. However, we noted the presence of variations in the form of different isomers and species-specific proteins. For example, two different galactosidase isomers were found in pathogenic *Burkholderia*, *α*-galactosidase (*B. pseudomallei*), and *β*-galactosidase (*B. ambifaria *MC40-6 and *B. cenocepacia *J2315). These different isomers affect sugar moieties found in the immunogenic capsular polysaccharide.

### 3.4. Unique Glycan-Associated Proteins in *B. pseudomallei* and *B. mallei*

Our comparative analysis had identified three unique proteins that are specific for *B. pseudomallei* (Table 2) and another six that are present only in *B. pseudomallei* and *B. mallei* (Table 3). Since these unique proteins were identified in well-characterized pathogenic members of the *Burkholderia *spp., two of these proteins were investigated further. These two proteins were chosen mainly because of their functions: (i) an *α*-galactosidase enzyme (BPSS2081) which is associated to the synthesis of sugar found in the immunogenic polysaccharide [59] and (ii) a putative surface protein (BPSS0796) that consists of a hemagglutinin motif which has been reported as important for bacterial adhesins [60].

### 3.5. *B. pseudomallei* Putative *α*-Galactose (*Bpss2081*)

The gene *bpss2081* encodes for an *α*-galactosidase that is needed for the synthesis of the *α*-galactose found in the immunogenic capsular polysaccharide of *B. pseudomallei *[59]. This sugar is located on the cell surface and may likely participate in host-pathogen interactions.

**Figure 2:**
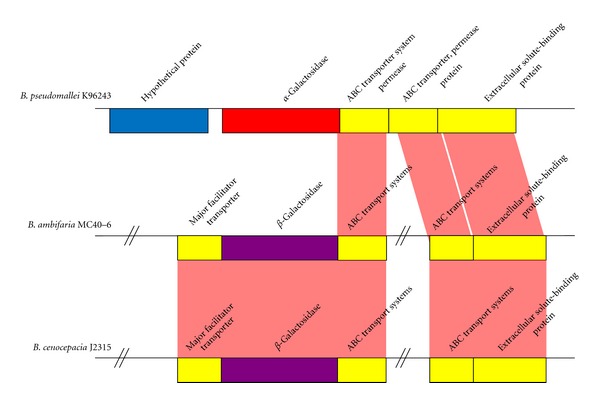
Structural comparison of galactosidase genes present in *B. pseudomallei* K96243, *B. ambifaria *MC40-6, and *B. cenocepacia *J2315. The Artemis Comparison tool (ACT) [45] was used to generate the data for this figure.

Comparative analysis revealed a significantly lower G+C content (53.3%) in the flanking gene of *B. pseudomallei *K96243 *bpss2081* compared to the G+C content (68.06%) of its own chromosomes. A previous study by Sim et al. [61] concluded that the Bp-like capsular polysaccharide (Bp-like CPS) was a foreign element recently acquired through horizontal gene transfer when they found that Bp-like CPS exhibited a lower G+C content (59.2%) compared to the *B. thailandensis* gene cluster. Using Sim et al. [61] as a reference, we conducted a comparison for the gene position relative to *bpss2081* in the remaining *Burkholderia* species and discovered that *B. ambifaria *MC40-6 and *B. cenocepacia *J2315 have another form of isomer, namely, *β*-galactosidase gene (Figure 2). The genes flanking *bpss2081* for *B. pseudomallei *K96243 was found to have a relatively lower G+C content (54.94%) compared to the *B. ambifaria *MC40-6 gene cluster (69.11%) and the *B. cenocepacia *J2315 gene cluster (65.58%) (Supplementary Table 2). This result is therefore consistent with the possibility that the *α*-galactosidase gene cluster in *B. pseudomallei* is a foreign element recently acquired through horizontal gene transfer. The magnitude of horizontally acquired genetic information is unexpectedly high in many bacterial pathogens [62, 63] and is by no means unique to *B. pseudomallei*.

Since most gene transfers take place between closely related organisms [64], we extended the investigation of *bpss2081 *to the closest homologs outside the *Burkholderia *genus. A BLASTP search revealed that the closest available homologs were from *Leptothrix cholodnii *(GenBank: YP_001792269.1) and *Curvibacter *putative symbiont of *Hydra magnipapillata *(GenBank: CBA30649.1) and belong to the order Burkholderiales. These three species share a common environmental niche; *B. pseudomallei *has been found in water supplies [65–67] while *L. cholodnii *resides in lowly running and metal-rich aquatic environments [68] and *H. magnipapillata *is a fresh water polyp. Due to similar or shared habitats, it is possible *bpss2081* may have been acquired by *B. pseudomallei* from other bacteria within the same environmental niche.

### 3.6. *B. pseudomallei* and *B. mallei* Putative Hemagglutinins

**Figure 3:**
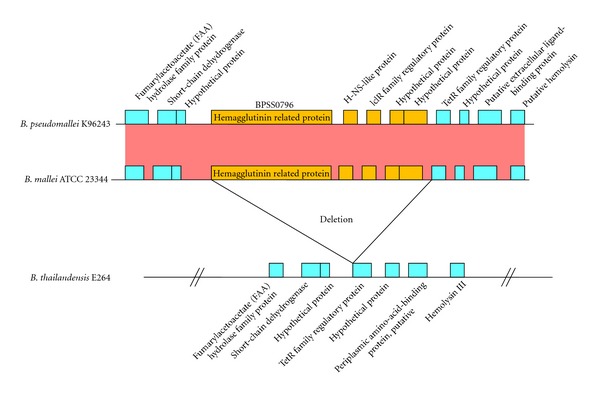
Structural comparison of surface-associated gene (*bpss0796*) in *B. pseudomallei *K96243, *B. mallei *ATCC 23344, and *B. thailandensis *E264. The genes (orange color boxes) indicate the deleted genes.

BPSS0796 is a surface protein with a hemagglutinin motif that was identified using the Pfam database [40]. Hemagglutinins are known glycoproteins in bacteria [60, 69, 70] and influenza viruses [71, 72]. The protein coded for by the structural gene *bpss0796* is among the four proteins unique to *B. pseudomallei *and *B. mallei*. Analysis of the surrounding genes indicates that the gene cluster *bpss0796-bpss0799* in *B. pseudomallei* K96243 and *bmaa0649-bmaa0653* in *B. mallei* ATCC 23344 do not have orthologs present in *B. thailandensis *(Figure 3). This absence provided further evidence that the corresponding region in *B. thailandensis *E264 had been deleted in comparison to the two pathogenic *Burkholderia *species, *B. pseudomallei *and *B. mallei. *Due to the absence of this gene cluster in *B. thailandensis, *the deletion event is believed to have only happened after the divergence of *B. thailandensis* from *B. pseudomallei *and *B. mallei* approximately 47 million years ago [73]. The structural comparison is not shown for the orthologs of *bpss0796* that are also absent in the other remaining *Burkholderia* species.

### 3.7. Significantly Reactive Glycan-Associated Proteins

We were also able to identify 5 serodiagnostic and cross-reactive antigens by cross referencing our data with the studies conducted by Felgner et al. [43] and Tiyawisutsri et al. [44] (Table 4). In the work conducted by Felgner et al. [43], BPSL1705 was found to be a serodiagnostic antigen that exhibited significantly different reactivity levels between the Singapore melioidosis positive and negative cohorts, while BPSL1902, BPSS2053, and BPSS1434 are cross-reactive antigens that are equally reactive in both melioidosis positive and negative cohorts. In another study conducted by Tiyawisutsri et al., the ortholog of BPSS1439 in *B. mallei* exhibited a strong antibody response during experimental glanders. Further analysis revealed one of these five to be an S-layer protein (BPSL1902).

### 3.8. A *B. pseudomallei* S-Layer Protein (BPSL1902)

There has yet to be an experimentally verified S-layer protein identified in the order Burkholderiales. This task is further complicated by the fact that there are no available overall sequence alignment profiles of the experimentally verified S-layer genes because sequence similarity only exists between S-layer protein genes of related species [74]. We first assigned BPSL1902, which was originally annotated as a putative membrane protein, to be an S-layer protein after analyzing *B. pseudomallei* protein sequences using the PSORTb subcellular location prediction program [49].

**Figure 4:**
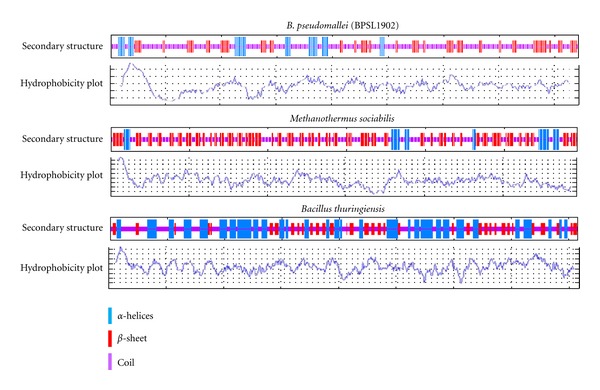
Composite diagram of comparisons for hydrophobicity plots and secondary structure predictions of *B. pseudomallei* S-layer protein (BPSL1902) versus *Bacillus thuringiensis ctc* (GeneBank ID: AJ012290) and *Methanothermus sociabilis slgA* (GeneBank ID: X58296) S-layer proteins. The program GOR IV [54] was used to generate the secondary structure while ProtScale was used to generate the Kyte and Doolittle plots [52].

BPSL1902 was then subjected to further analyses that revealed similar sequence features to experimentally verified S-layer proteins in other prokaryotes. Most S-layer proteins are in the weakly acidic pH range [75] and this is consistent with BPSL1902 which has a ProtParam [51] predicted isoelectric point of 4.58. The hydrophobicity plot of BPSL1902 showed a similar hydrophobicity profile to known S-layer proteins (Figure 4) where the transmembrane *α*-helices segments are arranged at the highly hydrophobic N-terminal. It is known to be common for S-layer proteins to have *α*-helices in the N-terminal region [75] while there were no other consistencies found in the form of secondary structure to other S-layer proteins in the remaining part of the sequences. As with other S-layers, the highly hydrophobic N-terminal *α*-helices most likely serve as an anchor to the membrane while the remaining hydrophilic regions are likely to protrude outwards from the membrane-attached section. The BPSL1902 S-layer was also subjected to EnsembleGly [50] glycosylation sites prediction and was found to potentially be highly O-glycosylated at the serine and threonine residues.

### 3.9. Features of the Glycosylated Regions

**Figure 5:**
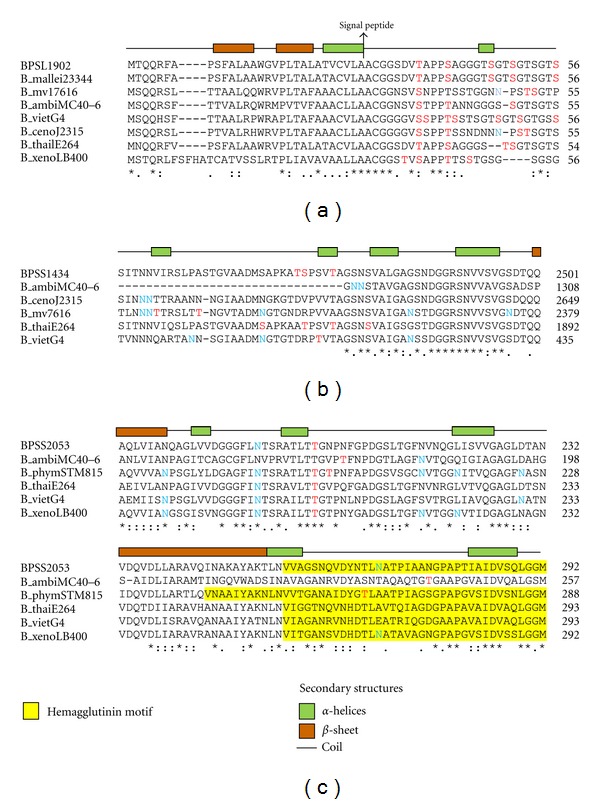
Multiple sequence alignment showed glycosylated amino acid in (a) BPSL1902, (b) BPSS1434, and (c) BPSS2053 with its respective orthologs. The amino acids in red represent O-glycosylation; blue color represents N-glycosylation and secondary structures shown are *B. pseudomallei* K96243 secondary structures. *Note.* BPSL1902, BPSS1434, and BPSS2053 are *B. pseudomallei* K96243 protein, B_mallei23344 is *B. mallei* ATCC 23344, B_mv17616 is *B. multivorans* ATCC 17616, B_ambifariaMC40-6 is *B. ambifaria* MC40-6, B_vietG4 is *B. vietnamiensis* G4, B_cenoJ2315 is *B. cenocepacia *J2315, B_thailE264 is *B. thailandensis* E264, and B_xenoLB400 is *B. xenovorans* LB400.

The significantly reactive *B. pseudomallei* surface proteins and their corresponding orthologs (Table 5) were subjected to multiple sequence alignment and EnsembleGly [50] glycosylation sites predictions. The analysis showed that conserved N- and C-terminal regions of protein sequences have a relatively low tendency to be glycosylated compared to the highly variable nonconserved regions. In the nonconserved regions, the propensity for glycosylation is indeed much higher (Figure 5) whereby N- and O-glycosylated residues tend to scatter along protein sequences and displaying inconsistency in the distribution of glycosylated residues. Hence this indicates that the nonconserved region is crucial in generating the versatility of glycosylation patterns as propensity for glycosylation seems to be high in this region. This is mainly believed to be due to the fact that the host immune system evasion mechanisms of pathogens often target the extremely variable and nonconserved portion of proteins [76].

### 3.10. Occurrence of Glycosylated Serine and Threonine Tandem Repeats That Are Potential Interfaces for Pathogen-Host Cell Interactions

**Figure 6:**
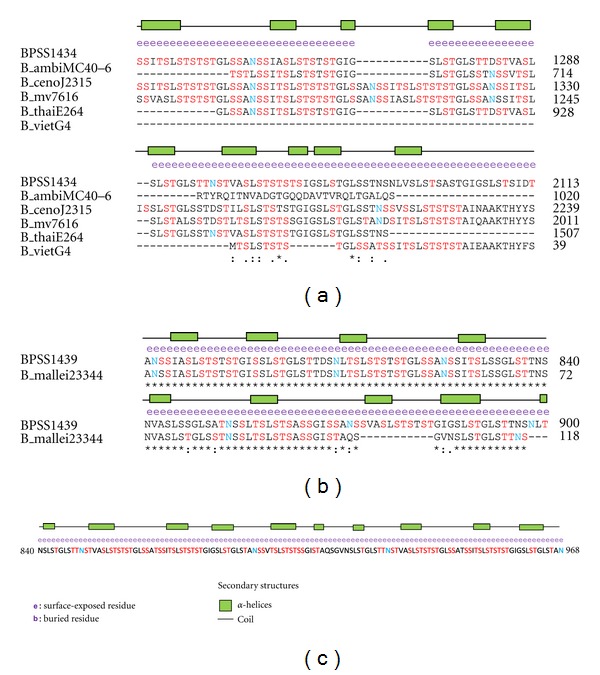
Glycosylated serine and threonine tandem repeats in (a) BPSS1434, (b) BPSS1439 and (c) BPSL1705 with its respective orthologs in *Burkholderia* spp. (if any). The amino acids in red represent O-glycosylation; blue color represents N-glycosylation. *Note.* BPSL1434, BPSL1705, and BPSS1439 are* B. pseudomallei* K96243 proteins, B_mv17616 is *B. multivorans* ATCC 17616, B_ambifariaMC40-6 is *B. ambifaria* MC40-6, B_vietG4 is *B. vietnamiensis* G4, B_thailE264 is *B. thailandensis* E264, and B_xenoLB400 is *B. xenovorans* LB400.

The glycosylation predictions carried out point to the possibility that BPSS1434, BPSL1705, and BPSS1439 consist of heavily glycosylated serine and threonine tandem repeats (STRs). The amino acid residues in STR regions were shown to be surface exposed and accessible to solvent with a high degree of glycosylation frequency (Figure 6). In this region, high surface accessibility will enable the amino acid with the glycan attached to protrude outwards and towards the host cell. This posture is important to allow for bacteria-host cell interaction because the surface glycans will form the first line of interaction to the mammalian host [77] in order to initiate infection [14, 15].

The presence of large amounts of glycosylated STRs is believed to contribute to host interaction considering that occurrence of tandem repeats (TRs) had been reported to have significant effects on pathogen adherence [78–81] and susceptibility to human diseases [82, 83]. Besides STRs, the presence of hemagglutinin motifs in BPSS1434, BPSS2053, BPSS1439, and BPSL1705 and hemagglutinin motif has been described to contribute to cell adherence and binding purposes. However, we observed that the glycosylation frequency in hemagglutinin motif was lower compared to the glycosylation frequency in the STRs region.

### 3.11. Selective Pressure Acting on Surface Proteins That Showed Significant Reactivity during Infection

The selective pressure acting on these proteins and their respective orthologs were then measured by calculating the number of nonsynonymous (amino acid changing) substitutions to synonymous (silent) substitutions rates, termed as *K*_*a*_ and *K*_*s*_, respectively. We found that the rates of synonymous substitution (*K*_*s*_) were much higher than nonsynonymous substitutions (*K*_*a*_) (Table 6) indicating that resulting amino acid sequences carry only a small number of amino acid changes. These proteins are therefore considered as conservative towards functional and structural changes.

It is often that when host immune systems coevolve with the bacterial pathogens, the surface proteins will undergo positive selection in an attempt to evade the host immune system and will have *K*_*a*_ > *K*_*s*_ [84–86]. This diversifying selection acts to change the protein in ways that increase prevalence of advantageous traits which is the driving force behind adaptive evolution [87].

However, given the high synonymous substitution rate (*K*_*s*_) of the proteins in Table 6, with their *K*_*a*_/*K*_*s*_ ratios being lower than 1, the amino acid diversity is therefore quite restricted. Due to this selective pressure (high *K*_*s*_) and the fact that these glycan-associated proteins are likely involved in interactions with the host due to their being located on the cell surface, successful bacterial pathogens would most likely have developed other strategies to circumvent host defense mechanisms. Therefore, this scenario leads to the possibility that one of the mechanisms to increase the adaptation of virulence-associated proteins lies behind the structurally diverse sugar moieties that binds to these proteins (shown with the presence of glycosylation sites). Furthermore, the lack of a clear delineation of a specific glycoprotein or glycan-associated protein to only pathogenic members of the *Burkholderia *spp. implies that while glycan-associated proteins may play roles in virulence, they are not involved directly in the pathogenesis mechanisms of the known *Burkholderia* pathogens.

## 4. Conclusions

Our use of this dataset has enabled us to contrast the genomes for members of the *Burkholderia *genus resulting in the identification of *Burkholderia *conserved orthologs and several proteins unique to pathogenic *Burkholderia *species. Further analysis of the data was also able to provide insights into the possible acquisition of species unique genes through gene transfer events. The selective measurement of the surface proteins signified that these proteins are quite conservative towards structural and functional changes unlike several other known surface proteins thereby implying a more prominent role played by the glycan components for diversification of the cell surface presentation to the host immune system.

## Supplementary Material

Supplementary Table 1 provides a listing of 178 glycans-associated protein found in *Burkholderia* spp. annotated and analysed in this study. Supplementary Table 2 presents the differences in G+C content for glycan associated proteins nalaysed from *B. pseudomallei* K96243, *B. ambifaria* MC40-6 and *B. cenocepacia* J2315.

## Figures and Tables

**Table 1:** The transposase found in position relatives to glycan associated gene cluster.

Gene clusters in *B. pseudomallei* K96243	Relative position in *B. mallei* ATCC 23344
*bpss1825-bpss1833* (exopolysaccharide biosynthesis-related tyrosine-protein kinase)	Transposase OrfA (bmaa0275), Transposase OrfB (bmaa0276)
*bpsl2769-bpsl2790* (capsular polysaccharide-related gene cluster)	Transposase OrfA (bma2284), Transposase OrfB (bma2283)
*bpsl1830-bpsl1834* (ribose synthesis)	Transposase OrfA (*bma1204*), Transposase OrfB (*bma1203*)

**Table 2:** Unique glycan associated proteins found in *B. pseudomallei* K96243.

GeneDB ID	Protein names	Subcellular location
BPSS2081	Putative *α*-galactosidase	Cytoplasmic
BPSL1705	Putative membrane protein	Outer membrane/extracellular
BPSS1215	Sugar transporter	Cytoplasmic membrane

**Table 3:** Unique glycan associated proteins found in *B.pseudomallei* K96243 and *B.mallei* ATCC 23344.

*B. pseudomallei* K96253	Protein names	Subcellular location	Orthologs in *B. mallei* ATCC 23344
BPSS0254	Putative ribose 5-phosphate isomerase	Cytoplasmic	YP_106366.1
BPSL2793	Putative D-glycero-d-manno-heptose 1,7-bisphosphate phosphatase	Cytoplasmic	YP_103855.1
BPSL2795	Putative sedoheptulose 7-phosphate	Cytoplasmic	YP_103857.1
BPSS0796	Putative surface-exposed protein	Outer membrane/extracellular	YP_105401.1
BPSS1727	Putative hemagglutinin related protein	Outer membrane	YP_106315.1
BPSS1439	Putative membrane-anchored cell surface protein	Outer membrane	YP_105520.1

**Table 4:** Surface glycan associated proteins that showed significant reactivity during infection.

GeneDB ID	Protein names	Subcellular location	Pathogenic description	Reference
BPSL1902	Putative membrane protein	S-Layer	Cross-reactive antigen	Felgner et al. [43]
BPSL1705	Putative membrane protein	Outer membrane/extracellular	Serodiagnostic antigen	Felgner et al. [43]
BPSS2053	Putative cell surface protein	Outer membrane	Cross-reactive antigen	Felgner et al. [43]
BPSS1434	Putative membrane-anchored cell surface protein	Outer membrane/extracellular	Cross-reactive antigen	Felgner et al. [43]
BPSS1439	Putative membrane-anchored cell surface protein	Outer membrane	Strong antibody response^U^	Tiyawisutsri et al. [44]

Serodiagnostic antigens: antigens that exhibit significantly different reactivity levels between the Singapore melioidosis positive and negative cohorts.

Cross-reactive antigens: antigens that are equally reactive in both melioidosis positive and negative individuals, healthy subjects from endemic or nonendemic areas, or patients with other infections.

^U^Strong antibody response: the experiment was carried out using the protein in *B. mallei* which is ortholog for BPSL1439. This protein in *B. mallei* exhibits strong antibody response during experimental glanders.

**Table 5:** Summary of significantly reactive surface proteins feature in *Burkholderia* spp. and the orthologs.

*B. pseudomallei* GeneDB ID	Glycosylation types	Additional features	Ortholog proteins	
			GenBank	Species
BPSL1902	N-linked;	—	YP_102742.1	*B. mallei* ATCC 23344
	O-linked		YP_443066.1	*B. thailandensis* E264
			YP_558210.1	*B. xenovorans* LB400
			YP_001579923.1	*B. multivorans* ATCC 17616
			YP_001808141.1	*B. ambifaria* MC40-6
			YP_001119324.1	*B. vietnamiensis* G4
			YP_002230652.1	*B. cenocepacia* J2315

BPSS1434	N-linked;	Serine threonine tandem repeats	YP_439154.1	*B. thailandensis* E264
	O-linked		YP_560399.1	*B. xenovorans* LB400
			YP_001583453.1	*B. multivorans* ATCC 17616
			YP_001815920.1	*B. ambifaria* MC40-6
			YP_001118486.1	*B. vietnamiensis* G4
			YP_001858800.1	*B. phymatum* STM815

BPSS2053	N-linked;	—	YP_443237.1	*B. thailandensis *E264
	O-linked		YP_557393.1	*B. xenovorans* LB400
			YP_001812417.1	*B. ambifaria* MC40-6
			YP_001116438.1	*B. vietnamiensis* G4
			YP_001861848.1	*B. phymatum* STM815

BPSS1439	N-linked;	Serine threonine tandem repeats	YP_105520.1	*B. mallei* ATCC 23344
	O-linked			

BPSL1705	N-linked;	Serine threonine tandem repeats	—	—
	O-linked			

**Table 6:** Nonsynonymous substitution (*K*_*a*_) and synonymous substitution (*K*_*s*_) rates between protein pairs and its orthologs as described.

Sequence 1	Sequence 2	Nonsynonymous substitution; *K*_*a*_	Synonymous substitution; *K*_*s*_	*K*_*a*_/*K*_*s*_
BPSS1439	*B.mallei* ATCC 23344 (Genbank: YP_105520.1)	0.0217786	0.160713	0.135513

BPSL1902	*B.mallei* ATCC 23344 (Genbank: YP_102742.1)	0.001901	0.014441	0.131671
	*B. thailandensis* E264 (Genbank: YP_443066.1)	0.022584	0.366201	0.061671
	*B. multivorans* ATCC 17616 (Genbank: YP_001579923.1)	0.178427	2.7173	0.065663
	*B. ambifaria* MC40-6 (Genbank: YP_001808141.1)	0.185665	1.8318	0.101357
	*B. vietnamiensis* G4 (Genbank: YP_001119324.1)	0.161194	1.62651	0.099104
	*B. cenocepacia* J2315 (Genbank: YP_002230652.1)	0.170878	2.93372	0.058246
	*B. xenovorans* LB400 (Genbank: YP_558210.1)	0.343855	3.80685	0.090325

BPSS2053	*B. thailandensis* E264 (Genbank: YP_443237.1)	0.366385	5.64031	0.064958
	*B. ambifaria* MC40-6 (Genbank: YP_001812417.1)	1.14359	3.06456	0.373167
	*B. vietnamiensis* G4 (Genbank: YP_001116438.1)	0.383681	4.61058	0.083218
	*B. xenovorans* LB400 (Genbank: YP_557393.1)	0.570889	5.6423	0.10118
	*B. phymatum* STM815 (Genbank: YP_001861848.1)	0.4192	2.5801	0.162475

BPSS1434	*B. thailandensis* E264 (Genbank: YP_439154.1)	0.091527	0.885211	0.103395
	*B. multivorans* ATCC 17616 (Genbank: YP_001583453.1)	0.360264	2.33222	0.154473
	*B. ambifaria* MC40-6 (Genbank: YP_001815920.1)	0.555408	5.0961	0.108987
	*B. vietnamiensis* G4 (Genbank: YP_001118486.1)	0.943879	5.11658	0.184474
	*B. xenovorans* LB400 (Genbank: YP_560399.1)	0.79562	5.64563	0.140927
	*B. phymatum* STM815 (Genbank: YP_001858800.1)	0.803449	5.62495	0.142837
