# Supplementary material for: Comparative Genome Sequence Analysis Reveals the Extent of Diversity and Conservation for Glycan-Associated Proteins in Burkholderia spp
Source: Comp Funct Genomics. 2012 Sep 6;2012:752867. doi: 10.1155/2012/752867 (PMC3443583; doi:10.1155/2012/752867)
Supplement: Supplementary file 1 — Supplementary Table 1 provides a listing of 178 glycans-associated protein found in Burkholderia spp. annotated and analysed in this study. Supplementary Table 2 presents the differences in G+C content for glycan associated proteins nalaysed from B. pseudomallei K96243, B. ambifaria MC40-6 and B. cenocepacia J2315. [file 752867.f1.pdf]

Additional Files

Additional File 1 - List of all conserved *B. pseudomallei* glycan associated proteins in nine *Burkholderia* spp.

* Proteins with homologs that are known essential genes

GeneDB ID	Description	*B. mallei* ATCC 23344	*B. thailandensis* E264	*B. multivorans* ATCC 17616	*B. ambifaria* MC40-6	*B. vietnamiensis* G4	*B. cenocepacia* J2315	*B. xenovorans* LB400	*B. phymatum* STM815
**BPSL0183***	penicillin-binding protein	YP_101998.1	YP_440701.1	YP_001581291.1	YP_001809743.1	YP_001121023.1	YP_002229641.1	YP_556651.1	YP_001856290.1
**BPSL0188***	aspartyl/glutamyl-tRNA amidotransferase subunit A	YP_102003.1	YP_440706.1	YP_001581286.1	YP_001809738.1	YP_001121018.1	YP_002229646.1	YP_556656.1	YP_001856295.1
**BPSL0189***	aspartyl/glutamyl-tRNA amidotransferase subunit B	YP_102004.1	YP_440707.1	YP_001581285.1	YP_001809737.1	YP_001121017.1	YP_002229647.1	YP_556657.1	YP_001856296.1
**BPSL0212***	S-adenosylmethionine synthetase	YP_104736.1	YP_440732.1	YP_001581259.1	YP_001809684.1	YP_001120992.1	YP_002229670.1	YP_556681.1	YP_001856319.1
**BPSL0262**	putative transglycosylase	YP_104784.1	YP_440791.1	YP_001581210.1	YP_001809637.1	YP_001120945.1	YP_002229716.1	YP_560785.1	YP_001859118.1
**BPSL0278**	peptidoglycan hydrolase	YP_104803.2	YP_440806.1	YP_001581193.1	YP_001809619.1	YP_001120928.1	YP_002229733.1	YP_560835.1	YP_001859172.1
**BPSL0312***	D-fructose-6-phosphate amidotransferase	YP_104837.1	YP_440846.1	YP_001580684.1	YP_001809584.1	YP_001120896.1	YP_002229771.1	YP_560775.1	YP_001859108.1
**BPSL0374**	metallo-beta-lactamase superfamily protein	YP_101939.1	YP_440905.1	YP_001578572.1	YP_001809529.1	YP_001120845.1	YP_002229831.1	YP_556801.1	YP_001856421.1
**BPSL0408**	penicillin-binding protein 6	YP_101907.1	YP_440938.1	YP_001578603.1	YP_001809498.1	YP_001120815.1	YP_002229862.1	YP_556834.1	YP_001856451.1
**BPSL0447***	NAD(P)H-dependent glycerol-3-phosphate dehydrogenase	YP_104680.1	YP_440978.1	YP_001578642.1	YP_001809463.1	YP_001120779.1	YP_002229900.1	YP_556875.1	YP_001856509.1
**BPSL0497**	putative phosphosugar-binding protein	YP_104649.1	YP_441006.1	YP_001578672.1	YP_001809434.1	YP_001120753.1	YP_002229936.1	YP_556898.1	YP_001856533.1
**BPSL0521***	ribose-phosphate pyrophosphokinase	YP_104604.1	YP_441032.1	YP_001578705.1	YP_001809413.1	YP_001120733.1	YP_002229957.1	YP_556919.1	YP_001856554.1
**BPSL0545***	excinuclease ABC subunit A	YP_104583.1	YP_441055.1	YP_001578728.1	YP_001809390.1	YP_001120710.1	YP_002229979.1	YP_556943.1	YP_001856578.1
**BPSL0603**	putative polysaccharide biosynthesis protein	YP_101880.1	YP_441077.1	YP_001584375.1	YP_001810774.1	YP_001117190.1	YP_002233951.1	YP_553083.1	YP_001862798.1
**BPSL0620**	glycosyl transferase group 1 protein	YP_101897.1	YP_441094.1	YP_001584374.1	YP_001810781.1	YP_001117211.1	YP_002233952.1	YP_557329.1	YP_001862801.1
**BPSL0667***	glycyl-tRNA synthetase subunit beta	YP_102052.1	YP_441141.1	YP_001578789.1	YP_001809312.1	YP_001120629.1	YP_002230057.1	YP_557038.1	YP_001856644.1
**BPSL0695**	putative ATP-dependent RNA helicase 1	YP_102079.1	YP_441164.1	YP_001578819.1	YP_001809288.1	YP_001120608.1	YP_002230087.1	YP_560338.1	YP_001856665.1

**BPSL0782**	putative type 4 fimbrial pilin protein	YP_102104.1	YP_441205.1	YP_001578848.1	YP_001809260.1	YP_001120583.1	YP_002230113.1	YP_560309.1	YP_001856684.1
**BPSL0791***	Putative ADP-heptose--LPS heptosyltransferase II	YP_102116.1	YP_441214.1	YP_001578857.1	YP_001809246.1	YP_001120574.1	YP_002230121.1	YP_560302.1	YP_001856692.1
**BPSL0798***	fructose-1,6-bisphosphate aldolase	YP_102124.1	YP_441221.1	YP_001578864.1	YP_001809239.1	YP_001120567.1	YP_002231941.1	YP_560295.1	YP_001856699.1
**BPSL0844**	putative ketopantoate reductase	YP_102166.1	YP_441263.1	YP_001578908.1	YP_001809194.1	YP_001117178.1	YP_002231896.1	YP_554561.1	YP_001862257.1
**BPSL0876***	3-deoxy-manno-octulosonate cytidylyltransferase	YP_103838.1	YP_441296.1	YP_001578941.1	YP_001809159.1	YP_001120467.1	YP_002231863.1	YP_560222.2	YP_001856774.1
**BPSL0878***	tetraacyldisaccharide 4'-kinase	YP_103836.1	YP_441298.1	YP_001578943.1	YP_001809157.1	YP_001120465.1	YP_002231861.1	YP_560220.1	YP_001856776.1
**BPSL1044**	alpha,alpha-trehalose-phosphate synthase	YP_104512.1	YP_441456.1	YP_001579088.1	YP_001808997.1	YP_001120316.1	YP_002231596.1	YP_557663.1	YP_001858366.1
**BPSL1048**	Putative sugar transport, integral membrane protein	YP_104515.1	YP_441459.1	YP_001579090.1	YP_001808995.1	YP_001120314.1	YP_002231594.1	YP_557664.1	YP_001858364.1
**BPSL1114**	putative ATP-dependent RNA helicase 2	YP_103512.1	YP_441535.1	YP_001579149.1	YP_001808930.1	YP_001120239.1	YP_002231536.1	YP_557740.1	YP_001858306.1
**BPSL1124**	putative LPS biosynthesis-related protein	YP_103500.1	YP_441545.1	YP_001579156.1	YP_001808922.1	YP_001120231.1	YP_002231526.1	YP_557744.1	YP_001858302.1
**BPSL1186**	glucose-1-dehydrogenase	YP_103460.1	YP_441589.1	YP_001579191.1	YP_001808886.1	YP_001120194.1	YP_002231493.1	YP_557793.1	YP_001858257.1
**BPSL1190**	putative glycosyltransferase	YP_103456.1	YP_441593.1	YP_001579195.1	YP_001808882.1	YP_001120190.1	YP_002231489.1	YP_557797.1	YP_001858253.1
**BPSL1209***	triosephosphate isomerase	YP_103436.1	YP_441612.1	YP_001579214.1	YP_001808863.1	YP_001120170.1	YP_002231470.1	YP_557816.1	YP_001858236.1
**BPSL1345**	putative exported transglycosylase protein	YP_102529.1	YP_443299.1	YP_001580214.1	YP_001807884.1	YP_001119086.1	YP_002230396.1	YP_559751.1	YP_001857106.1
**BPSL1358***	putative phosphoglucomutase	YP_102542.1	YP_443286.1	YP_001580203.1	YP_001807895.1	YP_001119097.1	YP_002230407.1	YP_559741.1	YP_001857116.1
**BPSL1402**	trigger factor	YP_103113.1	YP_442641.1	YP_001579533.1	YP_001808541.1	YP_001119694.1	YP_002231124.1	YP_558725.1	YP_001857245.1
**BPSL1404***	ATP-dependent protease ATP-binding subunit	YP_103111.1	YP_442644.1	YP_001579535.1	YP_001808539.1	YP_001119692.1	YP_002231122.1	YP_558727.1	YP_001857247.1
**BPSL1444**	putative sugar transferase	YP_103072.1	YP_442683.1	YP_001579573.1	YP_001808496.1	YP_001119649.1	YP_002231085.1	YP_558767.1	YP_001857285.1
**BPSL1471**	Putative polymixin resistance glucosyl transferase	YP_103047.1	YP_442713.1	YP_001579598.1	YP_001808471.1	YP_001119626.1	YP_002231060.1	YP_558634.1	YP_001857160.1
**BPSL1472***	putative LPS biosynthesis-related protein	YP_103046.1	YP_442714.1	YP_001579599.1	YP_001808470.1	YP_001119625.1	YP_002231059.1	YP_558634.1	YP_001857161.1
**BPSL1484***	ClpB heat-shock protein	YP_103036.1	YP_442726.1	YP_001579611.1	YP_001808458.1	YP_001119613.1	YP_002231047.1	YP_558648.1	YP_001857173.1
**BPSL1539**	putative fructokinase-like protein	YP_102977.1	YP_442781.1	YP_001579674.1	YP_001808396.1	YP_001119551.1	YP_002230982.1	YP_558686.1	YP_001857209.1
**BPSL1575**	putative sugar kinase	YP_102686.1	YP_442817.1	YP_001579711.1	YP_001808357.1	YP_001119516.1	YP_002230931.1	YP_559038.1	YP_001857482.1
**BPSL1909***	alpha-ketoglutarate decarboxylase	YP_102751.1	YP_443073.1	YP_001579932.1	YP_001808134.1	YP_001119317.1	YP_002230645.1	YP_558203.1	YP_001857949.1

**BPSL1918***	translation initiation factor IF-2	YP_102760.1	YP_443081.1	YP_001579940.1	YP_001808126.1	YP_001119309.1	YP_002230637.1	YP_558195.1	YP_001857957.1
**BPSL1945***	threonyl-tRNA synthetase	YP_102787.1	YP_443111.1	YP_001579964.1	YP_001808103.1	YP_001119282.1	YP_002230612.1	YP_559634.1	YP_001857786.1
**BPSL1972**	Putative antibiotic resistance membrane protein	YP_102662.1	YP_443140.1	YP_001580050.1	YP_001808074.1	YP_001119254.1	YP_002230587.1	YP_559659.1	YP_001857810.1
**BPSL1981***	UTP--glucose-1-phosphate uridylyltransferase	YP_102653.1	YP_443149.1	YP_001580059.1	YP_001808063.1	YP_001119245.1	YP_002230578.1	YP_559671.1	YP_001857820.1
**BPSL1994**	putative carbohydrate kinase	YP_102641.1	YP_443162.1	YP_001580076.1	YP_001808046.1	YP_001119230.1	YP_002230561.1	YP_559679.1	YP_001857828.1
**BPSL2104**	putative penicillin-binding protein	YP_103141.1	YP_442606.1	YP_001579506.1	YP_001808560.1	YP_001119712.1	YP_002231148.1	YP_559056.1	YP_001857497.1
**BPSL2129**	inositol-5-monophosphate dehydrogenase	YP_103165.1	YP_442580.1	YP_001579470.1	YP_001808591.1	YP_001119735.1	YP_002231190.1	YP_559301.1	YP_001857542.1
**BPSL2147***	UDP-N-acetylglucosamine acyltransferase	YP_103183.1	YP_442563.1	YP_001579454.1	YP_001808607.1	YP_001119751.1	YP_002231205.1	YP_559313.1	YP_001857554.1
**BPSL2149***	UDP-3-O-[3-hydroxymyristoyl] glucosamine N-acyltransferase	YP_103185.1	YP_442561.1	YP_001579452.1	YP_001808609.1	YP_001119753.1	YP_002231207.1	YP_559315.1	YP_001857556.1
**BPSL2153***	1-deoxy-D-xylulose 5-phosphate reductoisomerase	YP_103189.1	YP_442557.1	YP_001579448.1	YP_001808613.1	YP_001119757.1	YP_002231211.1	YP_559319.1	YP_001857560.1
**BPSL2185**	Family M22 non-peptidase homologue	YP_103222.1	YP_442525.1	YP_001579417.1	YP_001808644.1	YP_001119789.1	YP_002231242.1	YP_559353.1	YP_001857593.1
**BPSL2187**	putative ATP-dependent RNA helicase	YP_103224.1	YP_442523.1	YP_001579415.1	YP_001808646.1	YP_001119791.1	YP_002231244.1	YP_559355.1	YP_001857595.1
**BPSL2197***	glutamyl-tRNA synthetase	YP_103237.1	YP_442513.1	YP_001579405.1	YP_001808655.1	YP_001119800.1	YP_002231253.1	YP_559365.1	YP_001857605.1
**BPSL2270***	phosphopyruvate hydratase	YP_103309.1	YP_442424.1	YP_001579350.1	YP_001808714.1	YP_001120023.1	YP_002231306.1	YP_559434.1	YP_001857667.1
**BPSL2281***	lysyl-tRNA synthetase	YP_103320.1	YP_442413.1	YP_001579339.1	YP_001808725.1	YP_001120034.1	YP_002231317.1	YP_559446.1	YP_001857678.1
**BPSL2399**	putative glycosyltransferase	YP_102370.1	YP_442298.1	YP_001580316.1	YP_001807759.1	YP_001118935.1	YP_002230198.1	YP_554912.1	YP_001857415.1
**BPSL2408**	glycosyl transferase group 1 protein	YP_102360.1	YP_442289.1	YP_001580327.1	YP_001807749.1	YP_001118924.1	YP_002230188.1	YP_559760.1	YP_001857096.1
**BPSL2507**	cysteine synthase B	YP_102231.2	YP_442183.3	YP_001580431.1	YP_001807642.1	YP_001118822.1	YP_002232042.1	YP_560009.1	YP_001856989.1
**BPSL2509***	putative ADP-glycero-mannoheptose epimerase	YP_102234.1	YP_442181.1	YP_001580433.1	YP_001807640.1	YP_001118820.1	YP_002232044.1	YP_560011.1	YP_001856987.1
**BPSL2510***	putative ADP-heptose synthase	YP_102235.1	YP_442180.1	YP_001580434.1	YP_001807639.1	YP_001118819.1	YP_002232045.1	YP_560012.1	YP_001856986.1
**BPSL2511**	putative UDP-glucose dehydrogenase	YP_102236.1	YP_442179.1	YP_001580435.1	YP_001807638.1	YP_001118818.1	YP_002232046.1	YP_560013.1	YP_001856985.1
**BPSL2547***	fructose-1,6-bisphosphatase	YP_102278.1	YP_442142.1	YP_001580563.1	YP_001807596.1	YP_001118780.1	YP_002232104.1	YP_560077.1	YP_001856920.1
**BPSL2592**	lipopolysaccharide heptosyltransferase-1	YP_103680.1	YP_442116.1	YP_001580592.1	YP_001807568.1	YP_001118752.1	YP_002232132.1	YP_557616.1	YP_001856897.1
**BPSL2611**	maltose-binding protein	YP_103699.1	YP_442097.1	YP_001580612.1	YP_001807547.1	YP_001118730.1	YP_002232151.1	YP_557586.1	YP_001856864.1

**BPSL2614**	glucokinase	YP_103702.1	YP_442094.1	YP_001580615.1	YP_001807544.1	YP_001118727.1	YP_002232154.1	YP_557583.1	YP_001856861.1
**BPSL2663***	3-deoxy-D-manno-octulosonic-acid transferase	YP_103754.1	YP_442038.1	YP_001580675.1	YP_001807498.1	YP_001118676.1	YP_002232221.1	YP_557343.1	YP_001858485.1
**BPSL2665**	lipopolysaccharide heptosyltransferase-1	YP_103756.1	YP_442036.1	YP_001580677.1	YP_001807496.1	YP_001118674.1	YP_002232223.1	YP_557341.1	YP_001858487.1
**BPSL2666**	phosphoglucomutase	YP_1025421	YP_442035.1	YP_001580678.1	YP_001807495.1	YP_001118673.1	YP_002232224.1	YP_557340.1	YP_001858488.1
**BPSL2667**	Putative lipopolysaccharide biosynthesis protein	YP_103758.1	YP_442034.1	YP_001580679.1	YP_001807494.1	YP_001118672.1	YP_002232225.1	YP_557339.1	YP_001858489.1
**BPSL2668**	putative glycosyl transferase	YP_103759.1	YP_442033.1	YP_001580680.1	YP_001807493.1	YP_001118671.1	YP_002232226.1	YP_557338.1	YP_001858490.1
**BPSL2669**	putative glycosyl transferase	YP_103760.1	YP_442032.1	YP_001580681.1	YP_001807492.1	YP_001118670.1	YP_002232227.1	YP_557337.1	YP_001858491.1
**BPSL2670***	UDP-glucose 4-epimerase	YP_103761.1	YP_442031.1	YP_001580682.1	YP_001807491.1	YP_001118669.1	YP_002232228.1	YP_557336.1	YP_001858492.1
**BPSL2671**	putative undecaprenyl phosphate N-acetylglucosaminyltransferase	YP_103762.1	YP_442030.1	YP_001580683.1	YP_001807490.1	YP_001118668.1	YP_002232229.1	YP_557309.1	YP_001858509.1
**BPSL2672**	Putative epimerase/dehydratase polysaccharide-related biosynthesis protein	YP_103564.1	YP_442029.1	YP_001580685.1	YP_001807488.1	YP_001118667.1	YP_002232230.1	YP_557270.1	YP_001858510.1
**BPSL2681**	ABC transporter, ATP-binding component	YP_103572.1	YP_442020.1	YP_001580696.1	YP_001807479.1	YP_001121037.1	YP_002232239.1	YP_557252.1	YP_001858534.1
**BPSL2682**	putative ABC transporter, membrane permease	YP_103573.1	YP_442019.1	YP_001580697.1	YP_001807478.1	YP_001118663.1	YP_002232240.1	YP_557251.1	YP_001858535.1
**BPSL2683**	dTDP-4-dehydrorhamnose reductase	YP_103574.1	YP_442018.1	YP_001580780.1	YP_001807476.1	YP_001118655.1	YP_002232241.1	YP_557248.1	YP_001858537.1
**BPSL2684**	dTDP-6-deoxy-D-glucose-3,5 epimerase	YP_103575.1	YP_442017.1	YP_001580779.1	YP_001807475.1	YP_001118654.1	YP_002232242.1	YP_557250.1	YP_001858538.1
**BPSL2685***	glucose-1-phosphate thymidylyltransferase	YP_103576.1	YP_442016.1	YP_001580778.1	YP_001807474.1	YP_001118653.1	YP_002232243.1	YP_553601.1	YP_001858539.1
**BPSL2686***	dTDP-glucose 4,6-dehydratase	YP_103577.1	YP_442015.1	YP_001580777.1	YP_001807473.1	YP_001118652.1	YP_002232244.1	YP_557247.1	YP_001858540.1
**BPSL2697***	chaperonin GroEL	YP_103588.1	YP_442004.1	YP_001580709.1	YP_001807462.1	YP_001118641.1	YP_002232255.1	YP_557235.1	YP_001858554.1
**BPSL2826***	putative DnaJ chaperone protein	YP_103884.1	YP_441856.1	YP_001580813.1	YP_001807382.1	YP_001118560.1	YP_002232373.1	YP_557091.1	YP_001858715.1
**BPSL2827***	molecular chaperone DnaK	YP_103885.1	YP_441855.1	YP_001580814.1	YP_001807381.1	YP_001118559.1	YP_002232374.1	YP_557090.1	YP_001858716.1
**BPSL2844**	glycolate oxidase FAD binding subunit	YP_103966.1	YP_441837.1	YP_001580834.1	YP_001807362.1	YP_001118541.1	YP_002232393.1	YP_557072.1	YP_001858733.1
**BPSL2952***	glyceraldehyde 3-phosphate dehydrogenase 1	YP_104014.1	YP_441743.1	YP_001580928.1	YP_001807276.1	YP_001118459.1	YP_002232490.1	YP_560419.1	YP_001858816.1
**BPSL2967***	L-arabinose transporter ATP-binding protein	YP_104031.1	YP_441727.1	YP_001580944.1	YP_001807260.1	YP_001118444.1	YP_002232506.1	YP_560438.1	YP_001858832.1
**BPSL2968**	Putative L-arabinose transport	YP_104032.1	YP_441726.1	YP_001580945.1	YP_001807259.1	YP_001118443.1	YP_002232507.1	YP_560439.1	YP_001858833.1

	system, exported protein								
**BPSL2975***	Monofunctional biosynthetic peptidoglycan transglycosylase	YP_104039.1	YP_441719.1	YP_001580952.1	YP_001807252.1	YP_001118435.1	YP_002232514.1	YP_560445.1	YP_001858839.1
**BPSL2988**	putative sugar kinase	YP_104052.1	YP_441705.1	YP_001580965.1	YP_001807239.1	YP_001118422.1	YP_002232527.1	YP_560458.1	YP_001858852.1
**BPSL3025***	N-acetylglucosaminyl transferase	YP_104094.1	YP_441666.1	YP_001581017.1	YP_001807201.1	YP_001118382.1	YP_002232564.1	YP_560499.1	YP_001858891.1
**BPSL3030***	UDP-N-acetylmuramoylalanyl-D-glutamate--2,6-diami nopimelate ligase	YP_104099.1	YP_441661.1	YP_001581022.1	YP_001807196.1	YP_001118377.1	YP_002232569.1	YP_560504.1	YP_001858896.1
**BPSL3031***	peptidoglycan synthetase FtsI	YP_104100.1	YP_441660.1	YP_001581023.1	YP_001807195.1	YP_001118376.1	YP_002232570.1	YP_560505.1	YP_001858897.1
**BPSL3141***	UDP-N-acetylglucosamine 1-carboxyvinyltransferase	YP_104237.1	YP_443499.1	YP_001578518.1	YP_001807067.1	YP_001118249.1	YP_002229476.1	YP_560589.1	YP_001858979.1
**BPSL3215***	elongation factor Tu	YP_104181.1	YP_443587.1	YP_001578426.1	YP_001806978.1	YP_001118176.1	YP_002229399.1	YP_560673.1	YP_001859060.1
**BPSL3220***	DNA-directed RNA polymerase subunit beta'	YP_104174.1	YP_443579.1	YP_001578434.1	YP_001806986.1	YP_001118171.1	YP_002229394.1	YP_560678.1	YP_001859065.1
**BPSL3228**	elongation factor Tu	YP_104168.1	YP_443574.1	YP_001578426.1	YP_001806991.1	YP_001118163.1	YP_002229386.1	YP_560686.1	YP_001859073.1
**BPSL3273**	putative phosphosugar isomerase	YP_104316.1	YP_443641.1	YP_001578390.1	YP_001806934.1	YP_001118100.1	YP_002229327.1	YP_556740.1	YP_001856364.1
**BPSL3312**	putative glycosyltransferase	YP_104376.1	YP_443682.1	YP_001578351.1	YP_001806893.1	YP_001118059.1	YP_002229289.1	YP_560870.1	YP_001859196.1
**BPSL3319**	flagellin	YP_104383.1	YP_443690.1	YP_001578343.1	YP_001806886.1	YP_001118051.1	YP_002229280.1	YP_560880.1	YP_001859205.1
**BPSS0058**	excinuclease ABC subunit A	YP_104914.1	YP_438277.1	YP_001585503.1	YP_001815935.1	YP_001115649.1	YP_002153630.1	YP_558030.1	YP_001857777.1
**BPSS0145***	ATP-dependent RNA helicase DbpA	YP_106468.1	YP_438416.2	YP_001585202.1	YP_001809938.1	YP_001116350.1	YP_002232805.1	YP_556617.1	YP_001856255.1
**BPSS0218**	isomerase	YP_106403.1	YP_440365.1	YP_001583270.1	YP_001812039.1	YP_001116121.1	YP_002235289.1	YP_552396.1	YP_001860469.1
**BPSS0327**	sugar transport-related, membrane protein	YP_106030.1	YP_440260.1	YP_001583389.1	YP_001811859.1	YP_001116009.1	YP_002235110.1	YP_554433.1	YP_001860820.1
**BPSS0342***	sugar transport-related, membrane protein	YP_105552.1	YP_440244.1	YP_001583397.1	YP_001811852.1	YP_001116002.1	YP_002235103.1	YP_554419.1	YP_001862670.1
**BPSS0817**	Hypothetical protein macroglobulin	YP_105428.1	YP_439778.1	YP_001584065.1	YP_001811116.1	YP_001117502.1	YP_002234349.1	YP_552770.1	YP_001857177.1
**BPSS1219***	penicillin-binding protein	YP_105722.1	YP_439393.1	YP_001584350.1	YP_001810804.1	YP_001117219.1	YP_002233977.1	YP_553098.1	YP_001860001.1
**BPSS1577**	Putative cellulose synthase catalytic subunit [UDP-forming]	YP_106168.1	YP_438994.1	YP_001580105.1	YP_001808003.1	YP_001119200.1	YP_002230524.1	YP_553295.1	YP_001859460.1
**BPSS1580**	cellulose biosynthesis protein	YP_106171.1	YP_438990.1	YP_001580108.1	YP_001807999.1	YP_001119196.1	YP_002230520.1	YP_553292.1	YP_001859457.1
**BPSS1682***	UTP-glucose-1-phosphate uridylyltransferase	YP_106267.1	YP_438898.1	YP_001584575.1	YP_001810553.1	YP_001116909.1	YP_002233628.1	YP_558796.1	YP_001857312.1

**BPSS1684**	Putative lipopolysaccharide biosynthesys-related glycosyltransferase	YP_106269.1	YP_438894.1	YP_001584578.1	YP_001810550.1	YP_001116906.1	YP_002233626.1	YP_558793.1	YP_001857309.1
**BPSS1685**	Putative lipopolysaccharide biosynthesis related membrane protein	YP_106270.1	YP_438893.1	YP_001584579.1	YP_001810549.1	YP_001116905.1	YP_002233625.1	YP_558792.1	YP_001857308.1
**BPSS1688**	GCP-mannose 4,6-dehydratase	YP_106273.1	YP_438889.1	YP_001584582.1	YP_001810546.1	YP_001116902.1	YP_002233622.1	YP_558787.1	YP_001857304.1
**BPSS1689**	UDP-glucose 4-epimerase	YP_106274.1	YP_438888.1	YP_001584583.1	YP_001810545.1	YP_001116901.1	YP_002233621.1	YP_557246.1	YP_001858541.1
**BPSS1760***	O-sialoglycoprotein endopeptidase	YP_105141.1	YP_438816.1	YP_001584781.1	YP_001810461.1	YP_001116704.1	YP_002233531.1	YP_552520.1	YP_001860115.1
**BPSS2165**	hypothetical protein BPSS2165	YP_106481.1	YP_440429.1	YP_001583233.1	YP_001812080.1	P_001116163.1	YP_002235335.1	YP_555253.1	YP_001860293.1
**BPSS2167**	glycosyl transferase	YP_106483.1	YP_440430.1	YP_001583231.1	YP_001812082.1	YP_001116165.1	YP_002235337.1	YP_555254.1	YP_001860296.1
**BPSS2304***	penicillin-binding protein	YP_106567.1	YP_440511.1	YP_001584232.1	YP_001810924.1	YP_001117313.1	YP_002234102.1	YP_558824.1	YP_001860073.1
**BPSL2930**	Putative gluconate permease	YP_103991.1	YP_441766.1	YP_001580902.1	YP_001807296.1	YP_001118477.1	YP_002232467.1	YP_560394.1	YP_001858796.1
**BPSS1762***	1-deoxy-D-xylulose-5-phosphate synthase	YP_105137.1	YP_438814.1	YP_001584783.1	YP_001810459.1	YP_001116702.1	YP_002233529.1	YP_552518.1	YP_001860117.1
**BPSL2843**	Putative glycolate oxidase subunit GlcD	YP_103967.1	YP_441838.1	YP_001580833.1	YP_001807363.1	YP_001118542.1	YP_002232392.1	YP_557073.1	YP_001858732.1
**BPSL0839**	Putative carbohydrate kinase	YP_102161.1	YP_441258.1	YP_001578903.1	YP_001809199.1	YP_001120529.1	YP_002231901.1	YP_560257.1	YP_001856735.1
**BPSL0264**	Putative uncharacterized protein	YP_104786.1	YP_440793.1	YP_001581208.1	YP_001809635.1	YP_001120943.1	YP_002229718.1	YP_560783.1	YP_001859116.1
**BPSL2410**	Alpha,alpha-trehalose-phosphate synthase	YP_102358.1	YP_442285.1	YP_001580329.1	YP_001807747.1	YP_001118922.1	YP_002230184.1	YP_559763.1	YP_001857094.1
**BPSL0538**	Putative uncharacterized protein	YP_104589.1	YP_441048.1	YP_001578721.1	YP_001809397.1	YP_001120717.1	YP_002229972.1	YP_556935.1	YP_001856570.1
**BPSL2118**	Putative uncharacterized protein	YP_103151.1	YP_442591.1	YP_001579492.1	YP_001808576.1	YP_001119726.1	YP_002231167.1	YP_559091.1	YP_001857528.1
**BPSL2012***	Glutaminyl-tRNA synthetase	YP_102623.1	YP_443180.1	YP_001580094.1	YP_001808022.1	YP_001119213.1	YP_002230543.1	YP_559698.1	YP_001857142.1
**BPSL0313***	Bifunctional protein glmU	YP_104838.1	YP_440847.1	YP_001581157.1	YP_001809583.1	YP_001120895.1	YP_002229772.1	YP_560776.1	YP_001859109.1
**BPSL1115***	Glutamyl-Q tRNA(Asp) synthetase	YP_103510.1	YP_441536.1	YP_001579150.1	YP_001808929.1	YP_001120238.1	YP_002231535.1	YP_557741.1	YP_001858305.1
**BPSS0545**	Glutathione-independent formaldehyde dehydrogenase	YP_105240.1	YP_440064.1	YP_001583515.1	YP_001811689.1	YP_001115881.1	YP_002234932.1	YP_553725.1	YP_001859509.1
**BPSL0656**	Putative nucleotidyl transferase	YP_102041.1	YP_441130.1	YP_001578778.1	YP_001809324.1	YP_001120640.1	YP_002230046.1	YP_557025.1	YP_001856633.1
**BPSS0509**	Putative uncharacterized protein	YP_106327.1	YP_440102.1	YP_001584442.1	YP_001810697.1	YP_001117110.1	YP_002233861.1	YP_553101.1	YP_001859999.1
**BPSL1367***	Putative exopolyphosphatase	YP_102551.1	YP_443277.1	YP_001580194.1	YP_001807904.1	YP_001119106.1	YP_002230416.1	YP_559731.1	YP_001857124.1

**BPSL2315**	Endonuclease/exonuclease/phosphatase family protein	YP_103357.1	YP_442378.1	YP_001579313.1	YP_001808752.1	YP_001120061.1	YP_002231346.1	YP_559480.1	YP_001857706.1
**BPSS0882**	Putative uncharacterized protein	YP_105966.1	YP_439711.1	YP_001583918.1	YP_001811258.1	YP_001117684.1	YP_002234534.1	YP_553640.1	YP_001859445.1
**BPSL1413***	Glucose-6-phosphate isomerase	YP_103101.1	YP_442653.1	YP_001579542.1	YP_001808534.1	YP_001119687.1	YP_002231117.1	YP_558734.1	YP_001857254.1
**BPSS1424**	Putative AraC transcriptional regulatory protein	YP_105327.1	YP_439165.1	YP_001583463.1	YP_001811752.1	YP_001115923.1	YP_002235010.1	YP_553700.1	YP_001859647.1
**BPSL2608***	ABC transporter, ATP-binding component	YP_103697.1	YP_442100.1	YP_001580609.1	YP_001807550.1	YP_00111873.3	YP_002232148.1	YP_557589.1	YP_001856867.1
**BPSL2966**	L-arabinose transport system, permease protein	YP_104030.1	YP_441728.1	YP_001580943.1	YP_001807261.1	YP_001118445.1	YP_002232505.1	YP_560437.1	YP_001858831.1
**BPSS1581**	Endoglucanase	YP_106172.2	YP_438989.1	YP_001580109.1	YP_001807998.1	YP_001119195.1	YP_002230519.1	YP_553293.1	YP_001859458.1
**BPSL2630**	Putative lipoprotein	YP_103719.1	YP_442072.1	YP_001580626.1	YP_001807532.1	YP_001118717.1	YP_002232167.1	YP_560108.1	YP_001856851.1
**BPSL0829**	Putative periplasmic ABC transporter substrate-binding component	YP_102152.1	YP_441251.1	YP_001578894.1	YP_001809205.1	YP_001120535.1	YP_002231910.1	YP_560266.1	YP_001856728.1
**BPSL0509**	Glycosyl transferase family protein	YP_104617.1	YP_441019.1	YP_001578682.1	YP_001809422.1	YP_001120742.1	YP_002229948.1	YP_556909.1	YP_001856544.1
**BPSL2411**	Putative trehalose-phosphatase	YP_102357.1	YP_442284.1	YP_001580330.1	YP_001807746.1	YP_001118921.1	YP_002230183.1	YP_559764.1	YP_001857093.1
**BPSL0699**	Putative galactonate dehydratase protein	YP_102085.1	YP_441168.3	YP_001578823.1	YP_001809284.1	YP_001120604.1	YP_002230091.1	YP_560330.1	YP_001856672.1
**BPSL2609**	ABC transporter, membrane permease	YP_103698.1	YP_442099.1	YP_001580610.1	YP_001807549.1	YP_001118732.1	YP_002232149.1	YP_557588.1	YP_001856866.1
**BPSS1582**	Cyclic di-GMP binding protein	YP_106173.2	YP_438988.2	YP_001580110.1	YP_001807997.1	YP_001119194.1	YP_002230518.1	YP_553294.1	YP_001859459.1
**BPSL3419**	Putative GMC oxidoreductase	YP_104440.1	YP_443824.1	YP_001578274.1	YP_001806802.1	YP_001117950.1	YP_002229178.1	YP_560963.1	YP_001859265.1
**BPSL0312**	Glucosamine--fructose-6-phosphate aminotransferase [isomerizing] 1	YP_104837.1	YP_440846.1	YP_001581158.1	YP_001809584.1	YP_001120896.1	YP_002229771.1	YP_560775.1	YP_001859108.1
**BPSL3048***	Putative ribulose-phosphate 3-epimerase	YP_105303.1	YP_443414.1	YP_001581039.1	YP_001807178.1	YP_001118360.1	YP_002229567.1	YP_560522.1	YP_001858914.1
**BPSL1158***	Ribonuclease G	YP_103487.1	YP_441562.1	YP_001579165.1	YP_001808913.1	YP_001120220.1	YP_002231519.1	YP_557767.1	YP_001858295.1
**BPSL0321**	Putative uncharacterized protein	YP_104845.1	YP_440857.1	YP_001581149.1	YP_001809575.1	YP_001120887.1	YP_002229779.1	YP_556766.1	YP_001856388.1
**BPSS1714**	Putative AraC-family transcriptional regulator	YP_106302.1	YP_438865.1	YP_001584612.1	YP_001810516.1	YP_001116847.1	YP_002233591.1	YP_558828.1	YP_001862407.1
**BPSL2424**	Putative beta-hexosaminidase	YP_102344.1	YP_442270.1	YP_001580347.1	YP_001807726.1	YP_001118904.1	YP_002231960.1	YP_559908.1	YP_001857081.1
**BPSL0496**	Putative N-acetylglucosamine-	YP_104648.1	YP_441005.1	YP_001578671.1	YP_001809435.1	YP_001120754.1	YP_002229935.1	YP_556897.1	YP_001856532.1

	6-phosphate deacetylase								
**BPSL1844**	Nod factor export ATP-binding protein I	YP_102897.1	YP_443006.1	YP_001579826.1	YP_001808215.1	YP_001119390.1	YP_002230775.1	YP_558550.1	YP_001857862.1
**BPSL1871***	Ribose-5-phosphate isomerase A	YP_102931.1	YP_443035.1	YP_001579868.1	YP_001808174.1	YP_001119355.1	YP_002230686.1	YP_558486.1	YP_001857921.1
**BPSL2977**	Putative exoribonuclease II	YP_104041.1	YP_441717.1	YP_001580954.1	YP_001807250.1	YP_001118433.1	YP_002232516.1	YP_56044.7	YP_001858841.1
**BPSL2195**	Putative AraC-family transcriptional regulator	YP_103234.1	YP_442515.1	YP_001579407.1	YP_001808653.1	YP_001119798.1	YP_002231251.1	YP_559363.1	YP_001857603.1
**BPSS0872**	AraC family regulatory protein	YP_102055.1	YP_560360.1	YP_001578793.1	YP_001809308.1	YP_001120625.1	YP_002230061.1	YP_560360.1	YP_001856647.1
**BPSL1282**	Putative exodeoxyribonuclease V gamma chain	YP_103385.1	YP_443364.1	YP_001580271.1	YP_001807804.1	YP_001118970.1	YP_002230243.1	YP_558176.1	YP_001857977.1
**BPSL2971**	Putative galactonokinase	YP_104035.1	YP_441723.1	YP_001580948.1	YP_001807256.1	YP_001118440.1	YP_002232510.1	YP_560442.1	YP_001858836.1
**BPSL1107**	Putative uncharacterized protein	YP_103521.1	YP_441528.1	YP_001579142.1	YP_001808937.1	YP_001120246.1	YP_002231543.1	YP_557728.1	YP_001858317.1
**BPSL2613**	6-phosphogluconolactonase	YP_103701.1	YP_442095.1	YP_001580614.1	YP_001807545.1	YP_001118728.1	YP_002232153.1	YP_557584.1	YP_001856862.1
**BPSL2964**	Putative epimerase	YP_104028.1	YP_441730.1	YP_001580941.1	YP_001807263.1	YP_001118447.1	YP_002232503.1	YP_560434.1	YP_001858828.1
**BPSL1342***	Ribonuclease H	YP_102526.1	YP_443302.1	YP_001580217.1	YP_001807881.1	YP_001119083.1	YP_002230393.1	YP_559754.1	YP_001857103.1
**BPSS0776**	Putative uncharacterized protein	YP_105378.1	YP_439819.1	YP_001583185.1	YP_001812133.1	YP_001116287.1	YP_002235395.1	YP_560435.1	YP_001858829.1
**BPSL0076**	Ribonuclease P protein component	YP_104856.1	YP_443731.1	YP_001581337.1	YP_001809792.1	YP_001121136.1	YP_002229587.1	YP_561019.1	YP_001859314.1
**BPSS1864**	Putative AraC-family regulatory protein	YP_105054.1	YP_438713.1	YP_001578832.1	YP_001809271.1	YP_001120597.1	YP_002230099.1	YP_559963.1	YP_001857028.1
**BPSL0438**	Sugar transport PTS system IIa component	YP_104689.1	YP_440969.1	YP_001578633.1	YP_001809472.1	YP_001120788.1	YP_002229891.1	YP_556866.1	YP_001856499.1
**BPSL2673**	Putative undecaprenyl phosphate N-acetylglucosaminyltransferase	YP_103565.1	YP_442028.1	YP_001580686.1	YP_001807487.1	YP_001118666.1	YP_002232231.1	YP_557269.1	YP_001858511.1
**BPSL1304***	Putative LacI-family transcriptional regulator	YP_104561.1	YP_443340.1	YP_001580255.1	YP_001807824.1	YP_001118989.1	YP_002230264.1	YP_559537.1	YP_001857728.1
**BPSL3368**	Putative AraC-family transcriptional regulator	YP_104490.1	YP_443742.1	YP_001578326.1	YP_001806854.1	YP_001118000.1	YP_002229230.1	YP_556613.1	YP_001856253.1
**BPSL3408***	Glucose-inhibited division protein A	YP_104449.1	YP_443812.1	YP_001578285.1	YP_001806812.1	YP_001117960.1	YP_002229188.1	YP_560951.1	YP_001859255.1
**BPSL2145***	Ribonuclease HII	YP_103181.1	YP_442565.1	YP_001579456.1	YP_001808605.1	YP_001119749.1	YP_002231203.1	YP_559311.1	YP_001857552.1
**BPSL2452***	Putative ribonuclease E	YP_102317.1	YP_442242.1	YP_001580375.1	YP_001807698.1	YP_001118876.1	YP_002231988.1	YP_559937.1	YP_001857053.1
**BPSS1578**	Putative exported protein	YP_106169.1	YP_438993.1	YP_001580106.1	YP_001808002.1	YP_001115655.1	YP_002230523.1	YP_553296.1	YP_001859461.1

Additional File 2 - Calculation of G+C content in *B. pseudomallei* 96243, *B. ambifaria* MC40-6 and *B. cenocepacia* J2315.

a) Gene clusters in *B. pseudomallei* 96243

GeneDB ID	Protein names	Length(bp)	GC content (%)
BPSS2080	hypothetical protein	2034	46.95
BPSS2081	alpha-galactosidase	2172	57.73
BPSS2082	ABC transporter system permease	852	53.63
BPSS2083	ABC transporter, permease protein	954	54.93
BPSS2084	extracellular solute-binding protein	1242	53.3
BPSS2085	ABC transport system, ATP-binding protein	1245	58.15
BPSS2086	hypothetical protein	2061	57.35
BPSS2087	LacI family regulatory protein	1059	57.51
	**Average**	**1452**	**54.94**

b) Gene clusters in *B. ambifaria* MC40-6

GeneDB ID	Protein names	Length(bp)	GC content (%)
BamMC406_6026	binding-protein-dependent transport systems inner membrane component	852	67.25
BamMC406_6027	Beta-galactosidase	1971	69.96
BamMC406_6028	major facilitator transporter	1296	72.07
BamMC406_6029	arabinogalactan endo-1,4-beta-galactosidase	1173	67.52
BamMC406_6030	aldose 1-epimerase	1089	68.78
BamMC406_6031	AraC family transcriptional regulator	1194	68.01
BamMC406_6032	xylose isomerase	1323	66.97
BamMC406_6033	xylulokinase	1479	72.35
	Average	1297	69.11

c) Gene clusters in *B. cenocepacia* J2315

GeneDB ID	Protein names	Length(bp)	GC content (%)
BCAM2806	putative sugar ABC transporter ATP-binding protein	1158	70.73
BCAM2807	putative beta-galactosidase	1986	69.74
BCAM2808	putative extracellular solute-binding protein	1260	65.56
BCAM2809	putative ABC transporter system permease	873	66.09
BCAM2810	putative ABC transporter system permease	852	64.44
BCAM2811	hypothetical protein	264	60.61
BCAM2812	LysR family regulatory protein	945	64.76
BCAM2813	hypothetical protein	405	62.72
	**Average**	**968**	**65.58**
